# In Vitro Hypoxia/Reoxygenation Induces Mitochondrial Cardiolipin Remodeling in Human Kidney Cells

**DOI:** 10.3390/ijms25116223

**Published:** 2024-06-05

**Authors:** Arvydas Strazdauskas, Sonata Trumbeckaite, Valdas Jakstas, Justina Dambrauskiene, Ausra Mieldazyte, Kristupas Klimkaitis, Rasa Baniene

**Affiliations:** 1 Laboratory of Biochemistry, Neuroscience Institute, Lithuanian University of Health Sciences, Sukileliu Av. 13, LT-50162 Kaunas, Lithuania; arvydas.strazdauskas@lsmu.lt (A.S.); sonata.trumbeckaite@lsmu.lt (S.T.); 2 Department of Biochemistry, Faculty of Medicine, Lithuanian University of Health Sciences, Eiveniu Str. 4, LT-50161 Kaunas, Lithuania; 3 Department of Pharmacognosy, Faculty of Pharmacy, Lithuanian University of Health Sciences, Sukileliu Av. 13, LT-50162 Kaunas, Lithuania; valdas.jakstas@lsmu.lt; 4 Laboratory of Biopharmaceutical Research, Institute of Pharmaceutical Technologies, Lithuanian University of Health Sciences, Sukileliu Av. 13, LT-50162 Kaunas, Lithuania; justina.dambrauskiene@lsmu.lt; 5 Department of Drug Chemistry, Faculty of Pharmacy, Lithuanian University of Health Sciences, Sukileliu Av. 13, LT-50162 Kaunas, Lithuania; 6 Faculty of Medicine, Medical Academy, Lithuanian University of Health Sciences, A. Mickeviciaus Str. 9, LT-44307 Kaunas, Lithuania; ausra.mieldazyte@lsmu.lt (A.M.); kristupas.klimkaitis@stud.lsmu.lt (K.K.)

**Keywords:** cardiolipin, hypoxia/reoxygenation, ischemia/reperfusion, mitochondria, kidneys, AKI

## Abstract

Renal ischemia/reperfusion is a serious condition that not only causes acute kidney injury, a severe clinical syndrome with high mortality, but is also an inevitable part of kidney transplantation or other kidney surgeries. Alterations of oxygen levels during ischemia/reperfusion, namely hypoxia/reoxygenation, disrupt mitochondrial metabolism and induce structural changes that lead to cell death. A signature mitochondrial phospholipid, cardiolipin, with many vital roles in mitochondrial homeostasis, is one of the key players in hypoxia/reoxygenation-induced mitochondrial damage. In this study, we analyze the effect of hypoxia/reoxygenation on human renal proximal tubule epithelial cell (RPTEC) cardiolipins, as well as their metabolism and mitochondrial functions. RPTEC cells were placed in a hypoxic chamber with a 2% oxygen atmosphere for 24 h to induce hypoxia; then, they were replaced back into regular growth conditions for 24 h of reoxygenation. Surprisingly, after 24 h, hypoxia cardiolipin levels substantially increased and remained higher than control levels after 24 h of reoxygenation. This was explained by significantly elevated levels of cardiolipin synthase and lysocardiolipin acyltransferase 1 (LCLAT1) gene expression and protein levels. Meanwhile, hypoxia/reoxygenation decreased ADP-dependent mitochondrial respiration rates and oxidative phosphorylation capacity and increased reactive oxygen species generation. Our findings suggest that hypoxia/reoxygenation induces cardiolipin remodeling in response to reduced mitochondrial oxidative phosphorylation in a way that protects mitochondrial function.

## 1. Introduction

Acute kidney injury (AKI) is a serious clinical syndrome that can be caused by various factors and is characterized by renal structural injury and a decrease in kidney function in a short period of time [1]. One of the major causes of AKI is acute tubular necrosis, which may be caused by renal ischemia [2]. Renal ischemia and reperfusion are inevitable in transplantation and other renal surgeries; therefore, protective therapies are necessary to ameliorate the injury [3,4,5]. The interruption of blood flow into the kidneys during ischemia/reperfusion means the interruption of oxygen metabolism within the renal cells. A lack of oxygen usually induces anaerobic metabolism in the cells; however, in some parts of the kidney, such as proximal tubules, this metabolic pathway is unable to meet the high energy demands of the kidneys. Moreover, a lack of ATP interrupts normal Na^+^, Ca^2+^, and K^+^ transport through the cellular membrane, which leads to calcium overload, the generation of reactive oxygen species, and the opening of mitochondrial permeability transition pores after reoxygenation or reperfusion, all of which leads to the loss of tubular epithelial cells [4,6,7]. 

Mitochondria play an important role in hypoxia/reoxygenation injury because they consume over 95% of all oxygen that reaches our cells in order to produce ATP through oxidative phosphorylation [8,9]. They are also known to produce large amounts of reactive oxygen species (ROS) during hypoxia/reoxygenation, which also amplifies the damage by inducing the nonspecific oxidation of mitochondrial and cellular proteins, DNA, and various phospholipids, especially cardiolipin [10,11,12].

Cardiolipin is a mitochondrial signature phospholipid with a unique structure and distinct chemical characteristics, which allow it to play many important roles in mitochondrial metabolism [13,14]. Cardiolipins are essential for mitochondrial cristae formation [15], the stabilization of electron transport chain complexes, and the formation of respirasomes [16,17]; cardiolipins are involved in protein synthesis [18], mitophagy [19], mitochondrial fission and fusion [20], and even cell apoptosis [21,22]. Cardiolipin contains four acyl chains, which makes it very diverse in terms of fatty acid composition. It has also been shown that this acyl composition is tissue-specific and that the dominant fatty acids contain 18 carbons with different numbers of double bonds, primarily linoleic (C18:2) and oleic (18:1) acids [23]. Alterations of mitochondrial cardiolipin levels or fatty acid compositions are associated with several pathologies, such as Barth syndrome [24], ischemia/reperfusion [15,25], diabetes and obesity [26,27], cardiomyopathy [28], and others.

It has been observed that ischemia/reperfusion causes a decrease in cardiolipin content and the oxidation and/or hydrolysis of cardiolipins in various tissues, such as the liver, heart, or brain [15,25,29,30,31]. It has also been shown that various pathological conditions, including ischemia/reperfusion, induce abnormal cardiolipin remodeling, which further exacerbates mitochondrial injury through increased ROS generation and impaired oxidative phosphorylation [26,32,33,34,35]. There is evidence that hypoxia/reoxygenation alone may impair mitochondrial function; however, the level of impairment depends on the tissue/cell type [11,36]. Little information is available about renal cardiolipins and their alterations during hypoxia/reoxygenation or ischemia/reperfusion. Therefore, in this study, we analyze cardiolipins in human renal proximal tubule epithelial cells and evaluate the effect of hypoxia/reoxygenation on alterations of cardiolipin content, mitochondrial function, and the gene expression of enzymes involved in cardiolipin metabolism.

## 2. Results

### 2.1. Hypoxia/Reoxygenation Reduces RPTEC Cell Proliferation

**Figure 1:**
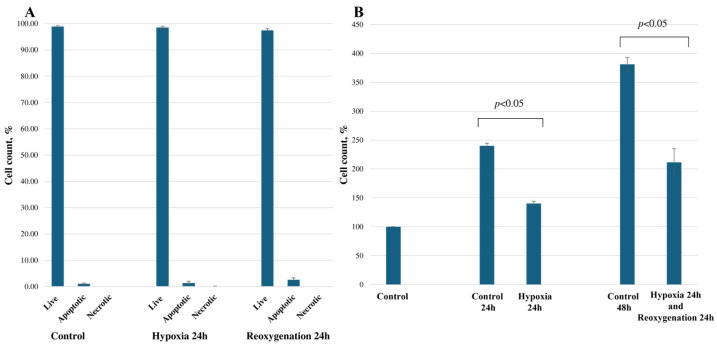
Effect of hypoxia and reoxygenation on RPTEC cell viability (**A**) and proliferation (**B**). Cell viability was assessed using fluorescent microscopy, where apoptotic and necrotic cells were observed. Cell proliferation was assessed by calculating the total number of cells in the hypoxia/reoxygenation and control groups with the respective amount of time for cultivation. The data are presented as the means of three independent experiments ± SE.

The interruption of oxygen metabolism in the kidneys leads to an induction of a multitude of pathologic mechanisms that eventually lead to cell death [12]. In this study, we induce 24 h of hypoxia in the cells by reducing the oxygen concentration in the environment to 2%, followed by 24 h of reoxygenation (O_2_ at 21%). Firstly, we assessed the effect of the hypoxia and reoxygenation model on RPTEC cell viability and proliferation. Fluorescent microscopy was used along with nuclei-specific Hoechst 33342 and propidium iodide dyes to analyze cell apoptosis and necrosis. We did not observe any significant changes in cell viability following hypoxia/reoxygenation; the number of live cells was well above 97% in all groups. After 24 h of reoxygenation, a slight increase in the number of apoptotic cells (from 1.08% to 2.53%) was noted; however, it was not statistically significant (Figure 1A). Then, we analyzed cell proliferation by calculating the total number of cells after hypoxia and after reoxygenation, along with the control groups that were cultivated for the same amount of time. We observed that hypoxia significantly decreased cell proliferation by 42%, and after reoxygenation, cell proliferation decreased by 45% compared to the respective control group (*p* < 0.05) (Figure 1B). These results show that our in vitro hypoxia/reoxygenation model does not cause extensive cell death but significantly reduces cell proliferation.

### 2.2. Hypoxia/Reoxygenation Reduces RPTEC Mitochondrial Respiration and Increases ROS Generation

**Figure 2:**
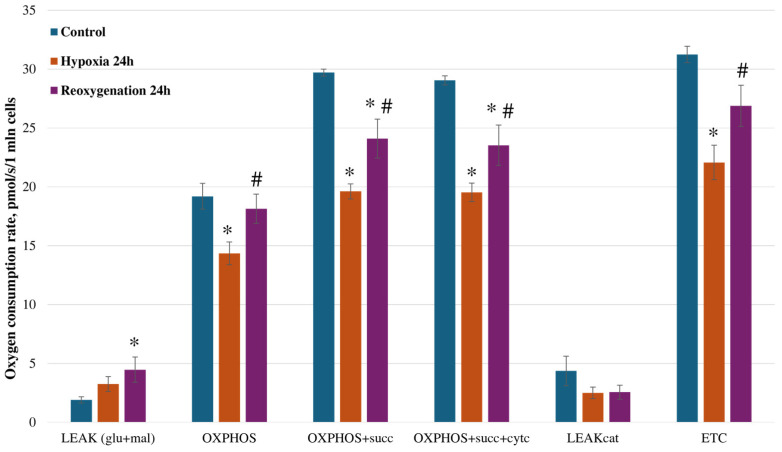
Effect of hypoxia and reoxygenation on RPTEC cell mitochondrial respiration. LEAK—proton leak state with the mitochondrial complex I substrates glutamate and malate; OXPHOS—oxidative phosphorylation state with ADP, and additionally, with succinate (succ) or cytochrome c (cytc); LEAKcat—proton leak state with the inhibitor adenine nucleotide carrier carboxyatractyloside (cat); ETC—electron transport state with the uncoupler dinitrophenol; The data are presented as the means of four independent experiments ± SE; * *p* < 0.05 compared to control; # *p* < 0.05 compared to hypoxia for 24 h.

Renal tubular epithelial cells are enriched in mitochondria due to high energy demand. Their primary source of energy is oxidative phosphorylation; therefore, fluctuations in oxygen concentration can impair mitochondrial functions and energy metabolism [37]. Therefore, we investigated whether hypoxia/reoxygenation affects RPTEC mitochondrial function. High-resolution respirometry was employed to analyze RPTEC mitochondrial respiration. The cells were permeabilized with digitonin, and the oxygen consumption rate at different mitochondrial respiration states was registered (Figure 2). After adding glutamate and malate, the substrates for mitochondrial complex I (NADH dehydrogenase) and oxygen consumption at the proton leak state (LEAK) were registered. We observed that 24 h of hypoxia caused a moderate 1.7-fold increase in oxygen consumption rate in the LEAK state, and after 24 h of reoxygenation, the oxygen consumption rate further increased to 2.34-fold that of the control (*p* < 0.05). After adding ADP (ATP synthase substrate), respiration at the phosphorylation state was registered (OXPHOS), and it was observed that 24 h of hypoxia significantly reduced the oxygen consumption rate in this respiration state by 25% compared to the control. After 24 h of reoxygenation, the oxygen consumption rate significantly increased by 26.6% compared to 24 h of hypoxia; however, it did not reach control levels. Then, succinate—a substrate for mitochondrial complex II (succinate dehydrogenase)—was added, and the maximal respiration rate in the OXPHOS state was registered. It was observed that 24 h of hypoxia decreased the oxygen consumption rate in this state by 32% (*p* < 0.05). After 24 h of reoxygenation, the oxygen consumption rate in this state increased by 23% compared to 24 h of hypoxia (*p* < 0.05); however, it remained 19% lower compared to the control levels (*p* < 0.05). Cytochrome c was added to check mitochondrial outer membrane permeability, but there were no alterations in the oxygen consumption rates in the OXPHOS state after hypoxia and reoxygenation. Carboxyatractyloside, an inhibitor of adenine nucleotide carrier, was added to measure the LEAK cat respiration rate and check inner membrane permeability; however, no significant changes in oxygen consumption rates were observed among the control, hypoxia, and reoxygenation groups. To analyze the maximal capacity of the electron transport chain, an uncoupler, dinitrophenol, was added, and oxygen consumption in the ETC respiration state was registered. It was observed that 24 h of hypoxia caused a 29% decrease in oxygen consumption in this respiratory state compared to the control (*p* < 0.05). After 24 h of reoxygenation, the oxygen consumption rate increased by 22% compared to 24 h of hypoxia (*p* < 0.05), but it was not restored to control levels. Thus, this shows that the mitochondrial respiratory chain might be affected during hypoxia/reoxygenation.

**Figure 3:**
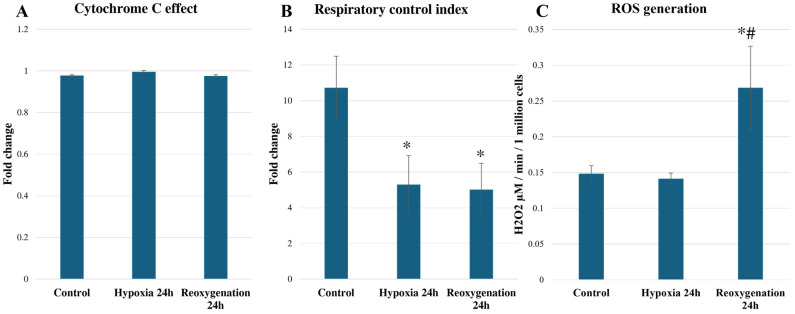
Effect of hypoxia and reoxygenation on RPTEC cell cytochrome c effect (**A**); respiratory control index (**B**) and H_2_O_2_ generation rate (**C**). The data are presented as the means of three–four independent experiments ± SE; * *p* < 0.05 compared to control; # *p* < 0.05 compared to hypoxia for 24 h.

In addition, the cytochrome c effect was calculated as a ratio of oxygen consumption rates after the addition of cytochrome c and before. A ratio > 1 shows that outer mitochondrial membrane permeability has increased. It was determined that hypoxia/reoxygenation did not alter the cytochrome c effect, which showed that mitochondrial outer membrane permeability was not affected (Figure 3A). However, an analysis of the respiratory control index (RCI) showed that both 24 h hypoxia and 24 h reoxygenation significantly decreased the phosphorylation capacity of mitochondria (Figure 3B). In addition, we observed that 24 h of reoxygenation significantly increased reactive oxygen species generation by 1.81 times in RPTEC cells (Figure 3C). In summary, these results show that in vitro hypoxia/reoxygenation significantly impairs mitochondrial function in terms of oxidative phosphorylation. 

### 2.3. Characterization of RPTEC Cardiolipins Using UPLC-MS/MS

**Figure 4:**
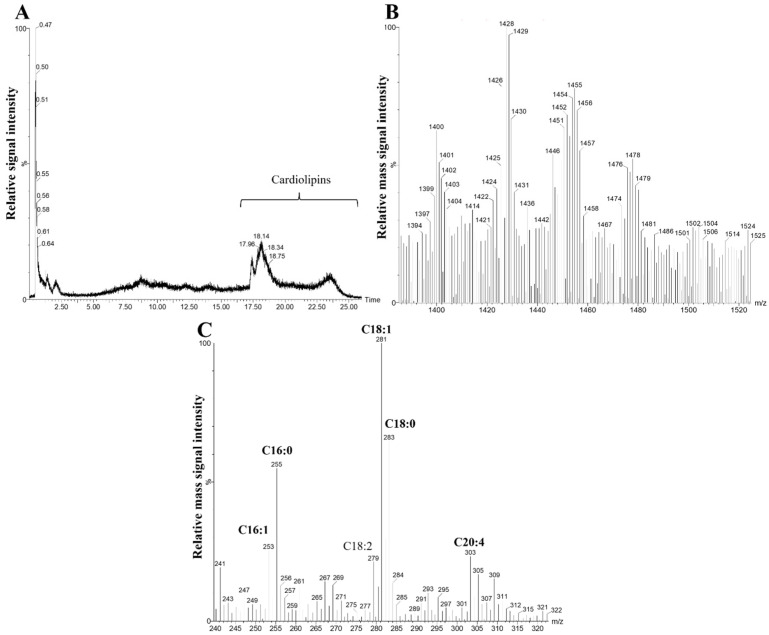
RPTEC lipid fraction analysis via UPLC-MS/MS, representative chromatogram, and mass spectra. (**A**) The total ion chromatogram (negatively charged lipid scan) of RPTEC lipid fractions; the mark “Cardiolipins” shows a region in the chromatogram where the RPTEC cell cardiolipins were eluted (approx. 17.50 to 26.00 min retention time); (**B**) total ion mass spectrum of negatively charged RPTEC lipids at 17.50–26.00 min of chromatographic retention time. A mass range of 1390–1525 *m*/*z* is presented, which belongs to different species of cardiolipins; (**C**) total ion mass spectrum of negatively charged RPTEC lipids at 17.50–26.00 min of retention time. A mass range of 240–322 *m*/*z* is presented, which belongs to the fatty acids of cardiolipins, and the more prominent masses of fatty acids are highlighted (C16:1, C16:0, C18:2, C18:1, C18:0, and C20:4).

Cardiolipins are essential structural and functional components in mitochondria. It has been shown that cardiolipin fatty acyl composition is specific to tissues and organs; however, there is still a lack of data about kidney cardiolipins [23]. In order to investigate cardiolipins in human RPTEC cells, total lipids were extracted from the cells and analyzed by reverse-phase ion pair chromatography and tandem mass spectrometry using an Acquity UPLC-MS/MS system. The composition of elution buffers allowed the cardiolipins, their fragments, and their separate fatty acids to become singularly charged (M − H^+^), and due to their large mass and relatively strong affinity for UPLC BEH C18 column particles, the cardiolipins were eluted late, starting at around the 18th minute of a 26 min-long elution (Figure 4A). Mass spectrometric analysis was performed in the negative ion scan mode, and it revealed four main mass clusters of cardiolipins, with masses ranging from 1394 to 1506 *m*/*z* (Figure 4B). Fatty acid composition analysis showed that cardiolipins contained mostly palmitic (C16:0), palmitoleic (C16:1), oleic (18:1), linoleic (C18:2), stearic (C18:0), and arachidonic (C20:4) fatty acids (Figure 4C). 

### 2.4. Hypoxia and Reoxygenation Increases Cardiolipin Amounts in RPTEC Cells

**Figure 5:**
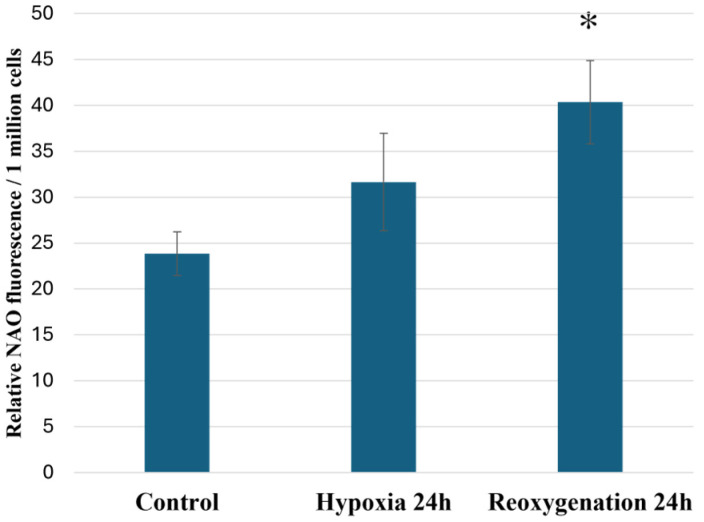
Effect of hypoxia and reoxygenation on the total amount of cardiolipin (NAO fluorescence) in RPTEC cells. The data are presented as the means of five–six independent experiments ± SE; * *p* < 0.05 compared to the control.

Before analyzing individual cardiolipin species in RPTEC cells, we evaluated the effect of hypoxia and reoxygenation on the total amount of cardiolipin by using a cardiolipin-specific fluorescent dye called nonyl acridine orange (NAO; acridine orange 10-nonyl bromide). The results showed that the total amount of RPTEC cardiolipin had a slight tendency to increase during 24 h of hypoxia; however, after 24 h of reoxygenation, it significantly increased to 1.69 times that of the control (*p* < 0.05) (Figure 5).

**Figure 6:**
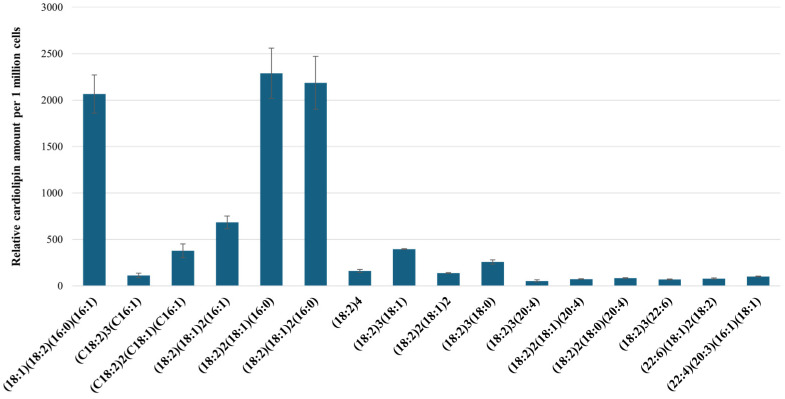
Relative abundance of 16 cardiolipin species in the RPTEC cell mitochondria of the control group. The labels on the x-axis represent the fatty acid composition of each individual cardiolipin form; the amounts of cardiolipin are expressed in relative units; the data are presented as the means of four independent experiments ± SE.

Next, we quantitated 16 cardiolipin forms out of the previously shown cardiolipin mass clusters (Figure 4B) and investigated the changes in their relative amounts after hypoxia and reoxygenation. We selected cardiolipins with masses as low as 1400 *m*/*z* CL (18:1)(18:2)(16:0)(16:1) and as high as 1502 *m*/*z* CL (22:6)(20:3)(16:1)(18:1). The most abundant forms of cardiolipins in the RPTEC cells were CL (18:1)(18:2)(16:0)(16:1), CL (18:2)_2_(18:1)(16:0), and CL (18:2)(18:1)_2_(16:0), with respective masses 1400, 1426, and 1428 *m*/*z*. The least abundant forms of cardiolipins were those with larger masses and contained arachidonic (C20:4) and docosahexaenoic (C22:6) fatty acids—CL (18:2)_3_(20:4), CL (18:2)_2_(18:1)(20:4), CL (18:2)_2_(18:0)(20:4), CL (18:2)_3_(22:6), and CL (22:6)(18:1)_2_(18:2), with respective masses of 1472, 1474, 1476, 1496, and 1500 *m*/*z* (Figure 6).

**Figure 7:**
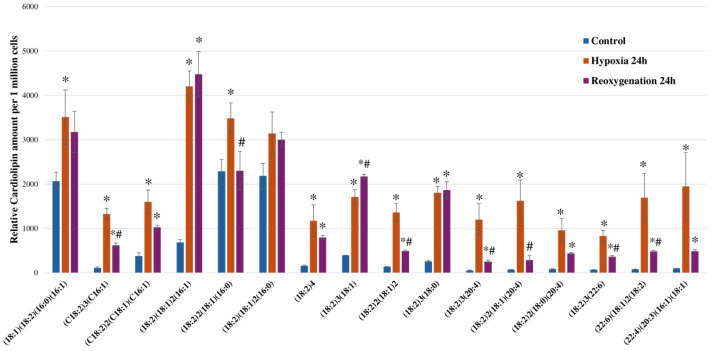
Effect of hypoxia and reoxygenation on the relative amounts of different forms of RPTEC cardiolipins. The amounts of cardiolipins are expressed as the chromatographic peak area standardized to an internal standard. The data are presented as the means of three–four independent experiments ± SE; * *p* < 0.05 compared to control; # *p* < 0.05 compared to hypoxia of 24 h.

We determined that 24 h of hypoxia substantially increased the amounts of cardiolipin across all examined forms (Figure 7). The amounts of cardiolipin species increased from 1.44 to 22.54 times after 24 h of hypoxia. The largest increase after 24 h of hypoxia was among those cardiolipins with longer fatty acids—CL (18:2)_3_(20:4), CL (18:2)_2_(18:1)(20:4), CL (22:6)(18:1)_2_(18:2), and CL (22:4)(20:3)(16:1)(18:1), which was 22.54, 22.16, 21.63, and 19.43 times higher than the control levels, respectively (*p* < 0.05). The least affected were the cardiolipin forms with a lower mass—CL (18:1)(18:2)(16:0)(16:1), CL (18:2)_2_(18:1)(16:0), and (18:2)(18:1)_2_(16:0); after 24 h of hypoxia, their amounts increased by 1.70 (*p* < 0.05), 1.52 (*p* < 0.05), and 1.44 times, respectively. Several cardiolipin forms further increased after 24 h of reoxygenation—CL (18:2)(18:1)_2_(16:1), (18:2)_3_(18:1), and (18:2)_3_(18:0), whereas the others decreased; however, these still remained higher than the control levels (*p* < 0.05). The most significant decrease after 24 h of reoxygenation compared to 24 h of hypoxia was found among the higher-mass cardiolipins—(18:2)_3_(20:4), (18:2)_2_(18:1)(20:4), (18:2)_2_(18:0)(20:4), (18:2)_3_(22:6), (22:6)(18:1)_2_(18:2), and (22:4)(20:3)(16:1)(18:1). 

### 2.5. Hypoxia and Reoxygenation Increases the Expression of Genes and the Respective Enzymes Involved in Cardiolipin Remodeling

**Figure 8:**
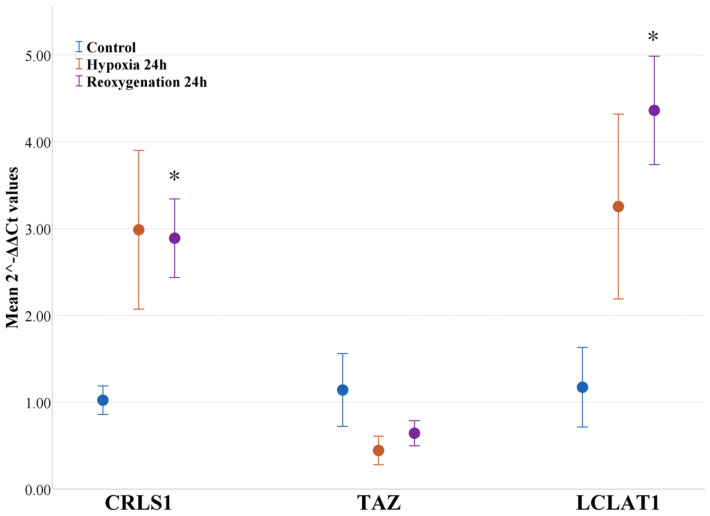
Effect of hypoxia and reoxygenation on the relative gene expression of cardiolipin synthesis and remodeling enzymes. Gene expression was normalized to *HPRT1* gene expression. *CRLS1*—cardiolipin synthase; *TAZ*—tafazzin; *LCLAT1*—lysocardiolipin acyltransferase 1. The data are presented as the means of three independent experiments ± SE; * *p* < 0.05 compared to control.

After determining that hypoxia and reoxygenation cause a significant increase in cardiolipins in RPTEC cells, we decided to check whether any of the cardiolipin synthesis or remodeling enzymes were affected at the level of gene expression and directly quantified the enzyme levels. The de novo synthesis of cardiolipins occurs in the inner mitochondrial membrane. This pathway does not produce tissue-specific cardiolipins, meaning that further fatty acid remodeling by several enzymes is required to form tissue-specific fatty acid composition in cardiolipins [17]. We analyzed the gene expression of cardiolipin synthase (*CRLS1*), the final enzyme in the cardiolipin de novo synthesis pathway, and two cardiolipin remodeling enzymes—lysocardiolipin acyl transferase (LCLAT1) and tafazzin (TAZ). After performing RT-qPCR, it was determined that two genes were transcribed more during hypoxia and reoxygenation in RPTEC cells—*CRLS1* and *LCLAT1* (Figure 8). The gene expression of cardiolipin synthase *CRLS1* was elevated 2.92 times after 24 h of hypoxia, although there was no statistical significance; however, the level remained elevated 2.82 times after 24 h of reoxygenation when compared to the control (*p* < 0.05). The gene expression of *LCLAT1* was elevated 2.77 times after 24 h of hypoxia, although not significantly, but after 24 h of reoxygenation, it was elevated even more— 3.72 times that of the control (*p* < 0.05). However, we also observed the slight inhibition of the tafazzin gene expression *TAZ*, even though it was not statistically significant; it decreased by 61% and 44% after 24 h of hypoxia and 24 h of reoxygenation, respectively. 

**Figure 9:**
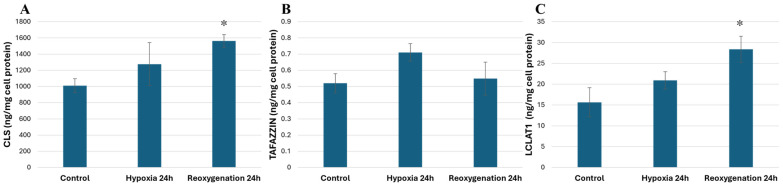
Effect of hypoxia and reoxygenation on cardiolipin synthesis and remodeling enzyme levels in RPTEC cells. (**A**) Cardiolipin synthase levels (CRLS1); (**B**) tafazzin levels; (**C**) lysocardiolipin acyltransferase 1 levels (LCLAT1). The data are presented as the means of three independent experiments ± SE; * *p* < 0.05 compared to the control.

Such gene expression results correspond to the amounts of the products of these genes (Figure 9). The results showed that after 24 h of hypoxia, there was a tendency for the amounts of cardiolipin synthase (CRLS1) and lysocardiolipin acyltransferase 1 (LCLAT1) to increase; however, after 24 h of reoxygenation, the amounts of these enzymes significantly increased to 1.54-fold and 1.81-fold, respectively, compared to the control (Figure 9A,C). However, hypoxia and reoxygenation did not have a significant effect on the levels of tafazzin in human RPTEC cells, although a slight increase after 24 h of hypoxia was observed (Figure 9B).

These results show that in vitro hypoxia/reoxygenation, indeed, induces effective cardiolipin resynthesis and remodeling in RPTEC cells, which substantially increases the amounts of cardiolipins.

## 3. Discussion

Renal ischemia/reperfusion is a complex pathophysiological process that encompasses many mechanisms of cellular and tissue injury, one of which is the interruption of normal oxygen metabolism, which is hypoxia and reoxygenation. Since mitochondria are primary oxygen consumers, their metabolism is substantially affected during ischemia/reperfusion—impaired electron transport, ATP production, ROS generation, the oxidation of mitochondrial lipids, increased ATP breakdown, and mitochondrial membrane permeabilization, all of which lead to further cellular damage and death [9,38,39]. Exclusive mitochondrial phospholipid cardiolipin, which plays many vital roles in mitochondrial homeostasis, is affected by oxidative stress during ischemia/reperfusion, and through oxidation and/or pathological remodeling, it may further exacerbate damage to mitochondria and the cell [31]. In this study, we employed an in vitro ischemia/reperfusion model by only manipulating the oxygen levels in the environment of the human renal proximal tubule epithelial cells (RPTEC). We show that in vitro hypoxia/reoxygenation reduced mitochondrial functions and substantially elevated cardiolipin content through the remodeling and resynthesis mechanisms in human kidney proximal tubule cells. 

Firstly, we show that the reduction of oxygen in a cellular environment for 24 h ended up reducing RPTEC cell proliferation; however, it did not affect cell death. Oxygen concentrations vary among different organs, and oxygen concentration in human kidney tissue is about 7% or 54 mmHg [40]. In our study, in vitro hypoxia was induced at 2% oxygen concentration; therefore, it could be said that such hypoxic conditions were mild to RPTEC cells and, therefore, did not cause significant cellular death. Nevertheless, similar results have also been observed even with lower oxygen concentrations in human synovial mesenchymal cells, where 5% or 0.5% oxygen concentrations did not have a significant effect on cell apoptosis [41]. There are many different in vitro ischemia/reperfusion models that induce the complete removal of oxygen in the cell culture environment (anoxia); the removal of nutrients, such as glucose, and amino and fatty acids, as well as leaving only physiological salt solutions mimics ischemic conditions [42,43,44]. Such in vitro ischemia/reperfusion conditions have been proven to induce significant cell death in human RPTEC cells [45], isolated mouse cardiomyocytes [46], and even in human cardiomyocyte models [47]. 

Mitochondria play an important role in ischemia/reperfusion injury. The lack of oxygen during ischemia inhibits oxidative phosphorylation and, therefore, substantially reduces energy generation, which disrupts the ATP-dependent processes in the cell, such as ion exchange. This leads to the accumulation of Ca^2+^ in the cytosol, which further triggers mitochondrial permeability transition, increased ROS generation during reperfusion, and more uncontrolled oxidation that eventually contributes to cellular death [48,49]. Our experimental study also showed that hypoxia significantly reduced mitochondrial respiration and oxidative phosphorylation capacity; however, it did not increase mitochondrial outer membrane permeability. During reoxygenation, we observed that respiration rates increased in almost all states; however, it did not reach the control levels. In addition, we also show that ROS generation increased during reoxygenation, which could have caused more damage to the mitochondria and, therefore, inhibited the complete restoration of mitochondrial respiration. However, a recent study showed that 1% O_2_ hypoxia induces mitochondrial protein lactylation, which negatively regulates oxidative phosphorylation in mouse muscle cells [50] and could be another mechanism that is involved in the inhibition of mitochondrial respiration in RPTEC cells during hypoxia since it was shown that renal mitochondrial proteins also undergo lactylation during ischemia/reperfusion in mice [51]. 

Ischemia and reperfusion inflict a number of damaging strikes on the mitochondria, including structural changes, such as cristae fragmentation [15], or the disruption of essential phospholipids, such as cardiolipin [52,53]. Cardiolipin, a distinctly shaped mitochondrial phospholipid, plays many vital roles not only in mitochondrial physiology but in pathophysiology as well, especially in ischemia/reperfusion injury [37]. Since cardiolipin interacts with electron transport chain complexes, it becomes an easy target for ROS-mediated peroxidation. Elevated ROS synthesis, or oxidative burst, is a hallmark of reperfusion; therefore, cardiolipin oxidation becomes inevitable and further aggravates mitochondrial dysfunction [31]. The oxidation of cardiolipin is not the only modification that exacerbates mitochondrial dysfunction: some time ago, it was revealed that the pathological remodeling of cardiolipin fatty acids contributes to mitochondrial damage in various conditions such as Barth syndrome, diabetes, aging, or heart diseases [26,28,33,54]. Our study shows that hypoxia/reoxygenation alone induces the mRNA expression of cardiolipin synthase and lysocardiolipin acyltransferase 1, a remodeling enzyme, along with their protein levels in RPTEC cells. This was also followed by a substantial increase in many cardiolipin species in these cells. Cardiolipin synthase is an enzyme that catalyzes the final step of de novo cardiolipin synthesis; however, it does not form a cardiolipin with a tissue-specific fatty acid composition. Therefore, after de novo synthesis, cardiolipins are remodeled by three enzymes—tafazzin, monolysocardiolipin acyltransferase, and lysocardiolipin acyl transferase 1 (LCLAT1, also known as acyl-CoA lysocardiolipin acyltransferase 1 or ALCAT1) [55,56]. It has been shown that ALCAT1 overexpression is involved in the pathological remodeling of cardiolipin, which impairs mitochondrial functions and leads to further cellular damage. This was recently shown in a study where overexpressed ALCAT1 increased cardiolipin oxidation and mitochondrial dysfunction in the kidneys of patients with diabetic kidney disease, as well as in mouse and cell models of such diseases [35]. Another study showed that ALCAT1 overexpression in C2C12 cells decreased C18 and C16 fatty acyl content in cardiolipins but increased the longer fatty acids C20 and C22 in cardiolipins [26]. In a recent study with a mouse model of myocardial infarction (MI), Jia et al. showed that MI upregulated ALCAT1 protein expression in the heart, which also caused a decrease in tetralinoleyl cardiolipin levels and induced cardiolipin remodeling by incorporating longer chain fatty acids. However, the ablation/inhibition of ALCAT1 in these mice reduced long-chain fatty acids in heart cardiolipins and restored tetralinoleyl cardiolipin levels as well after MI [34]. In our study, we also show that hypoxia/reoxygenation increased the mRNA levels of *LCLAT1* and *CLRS1* and the levels of the respective enzymes, which, in turn, increased the existing cardiolipin levels during hypoxia. In fact, the most significant increase was observed among cardiolipins with longer chain fatty acids (C20:4, C22:6, C22:4, C20:3). In addition, we showed that a substantial decrease in these species of cardiolipins happened after reoxygenation compared to hypoxia, and this happened along with a significant increase in ROS generation, which indicates that these highly unsaturated cardiolipin species could have undergone peroxidation. Indeed, many studies show that ischemia/reperfusion causes cardiolipin peroxidation, for example, in rat liver [10,25] or rat hearts [30,57,58]; however, we did not analyze oxidized cardiolipin forms in this study. Despite that, many cardiolipin species remained at significantly increased levels after reoxygenation compared to the control group. Allen et al. showed that ex vivo ischemia/reperfusion decreased the total amount of cardiolipins in mouse hearts; however, that was mainly caused by a decrease in tetralinoleyl cardiolipin, which comprised more than 70% of all investigated species, while many other minor species actually increased, which could have been a result of activated cardiolipin remodeling mechanisms [15]. A similar effect was observed in a study carried out by Martens et al., where they found that the total amount of cardiolipins in rat liver decreased after ischemia/reperfusion; however, a detailed analysis of individual cardiolipin species showed that about 70% of them, which were the minor species, had increased [25]. Wu et al. carried out a study using a retinal ischemia/reperfusion model with mice and discovered that 7 days after reperfusion, cardiolipin levels had decreased; in contrast, other lipids in the retina, such as phosphatidic acid, phosphatidylglycerol, diacylglycerol, sphingosines, sphingomyelins, ceramides, and others, substantially increased [53]. In our study, the total amount of cardiolipin (measured using NAO stain) and individual cardiolipin amounts increased after hypoxia/reoxygenation, which was catalyzed by increased amounts of cardiolipin synthase and LCLAT1. Since we only changed oxygen concentration in the cell environment, all nutrients present in the growth medium were still available to the cells; therefore, they were fully capable of synthesizing new cardiolipins and/or carrying out remodeling. Different in vitro ischemia/reperfusion conditions, such as nutrient deprivation or glycolysis inhibition, might have a different effect on cardiolipin alterations. 

## 4. Materials and Method

### 4.1. Cell Culture

Immortalized human renal proximal tubule epithelial cells (RPTEC/TERT1) were obtained from Evercyte GmbH (Vienna, Austria). The cells were cultured in 25 cm^2^ flasks at 37 °C in a 21% O_2_, 5% CO_2_ humidified atmosphere. The cell growth medium consisted of a 1:1 mixture of Dulbecco’s modified Eagle’s medium and Ham’s F-12 nutrient mix with 2 mM Glutamax (DMEM/F12, Glutamax; catalog No. 10565018, Gibco, Paisley, UK), supplemented with 10 µg/mL human epidermal growth factor, 25 µg/mL hydrocortisone, 6.7 µg/mL sodium selenite, 5 µg/mL transferrin, 10 µg/mL insulin, 100 µg/mL geneticin (G418), and 4% fetal bovine serum. Cells were subcultured at confluency of 95%, roughly every two to three days by trypsinization.

### 4.2. Induction of Hypoxia/Reoxygenation

To induce hypoxia/reoxygenation, cells were first seeded in regular culture flasks or 96-well plates at a density of 40,000 cells/cm^2^ and left overnight to allow cells to attach. For hypoxia, the cells were placed in Baker Ruskinn InVivo2 humidified hypoxia workstation (Baker Co., Bridgend, UK) at 37 °C in a 2% O_2_, 5% CO_2_ humidified atmosphere for 24 h. Cell culture medium was kept in the workstation overnight to equilibrate dissolved gases and was used to replace normal cell growth medium upon the induction of hypoxia in the workstation. For reoxygenation, after 24 h of hypoxia, the cells were removed from the workstation, growth medium was replaced with fresh medium, and the cells were put in an incubator at regular culturing conditions for 24 h. 

### 4.3. Cell Viability Assessment

Cell viability was assessed by fluorescent microscopy using Hoechst 33342 and propidium iodide staining at final concentrations of 20 µg/mL and 10 µg/mL, respectively. Cells in 96-well plates were incubated with both dyes for 15 min at 37 °C, then fixed with paraformaldehyde for 15 min at 37 °C. Cells were analyzed under an Olympus IX71S1F-3 fluorescent microscope. The total number of cells were calculated in at least five randomly selected microscopic fields at 200× magnification. Cells with PI-positive (red) nuclei were considered necrotic, cells with condensed and fragmented nuclei (Hoescht 33342, blue) were considered apoptotic, and cells with homogenously stained nuclei (Hoescht 33342, blue) were considered live. Quantification was carried out using ImageJ version 1.53.

### 4.4. Mitochondrial Respiration Assessment

Cell mitochondrial respiration analysis was performed using Oroboros Oxygraph-2k (Innsbruck, Austria) high resolution respirometer. Experiments were performed in duplicates in MiR05 respiration medium (EGTA 0.5 mM, MgCl_2_ 3 mM, lactobionic acid 60 mM, taurine 20 mM, KH2PO4 10 mM, HEPES 20 mM, D-sucrose 110 mM) at 37 °C under constant stirring at 750 rpm. The experiment started with the addition of 2 × 10^6^ cells to O2k chambers with 2 mL respiration medium and 5 mM glutamate and 7.5 mM malate, substrates for the electron transport chain complex I. Then cell membrane was permeabilized with 12 µM digitonin and LEAK (L) respiration state was registered. Then 2 mM ADP was added and OXPHOS (P) respiration state was registered. II complex substrate succinate 12.5 mM was added, and maximal respiration was registered in OXPHOS (P(Succ)) state. To assess outer mitochondrial membrane permeabilization 32 µM cytochrome c was added (P(CytC)). To assess inner mitochondrial membrane permeabilization 1 nM carboxyatractyloside was added (L(CAT)). Then maximal electron transport chain capacity was registered (E) in the presence of an uncoupler 45 nM dinitrophenol. Non-mitochondrial oxygen consumption rate was recorded after adding 10 mM azide, IV complex inhibitor. Respiration values were normalized to the total number of cells. 

### 4.5. Analysis of H_2_O_2_ Production

H_2_O_2_ production was measured using Amplex Red Assay. RPTEC cells were collected by trypsinization, calculated using Trypan Blue and resuspended in HBSS solution with 30 µg/mL horseradish peroxidase and 15 µM Amplex Red at 7.3 × 10^5^ cells/mL. Cell suspension was placed in black plastic 96-well plate. The rate of H_2_O_2_ production was analyzed for 30 min by continuous measurement of fluorescence in microplate fluorescence reader Fluoroscan Ascent (Thermo Scientific, Waltham, MA, USA) with excitation 544 nm and emission 590 nm. Fluorescence signal was calibrated using known concentrations of H_2_O_2_, and the results were expressed as the amount of H_2_O_2_ µM per minute per 1 million cells.

### 4.6. Total Cardiolipin Content Fluorometric Analysis

Total cardiolipin amount was analyzed using cardiolipin-specific fluorescent stain nonylacridine orange (NAO; acridine orange 10-nonyl bromide). RPTEC cells were collected by trypsinization, calculated using Trypan Blue staining and resuspended in culture medium with 1 µM NAO at 1 million/mL. Cell suspension was incubated for 30 min at 37 °C in constant stirring. After incubation cells were centrifuged, resuspended in PBS at 1 million/mL and placed in black plastic 96-well plates. NAO fluorescence was immediately measured using microplate fluorescence reader Fluoroscan Ascent (Thermo Scientific) with excitation 485 nm, emission 538 nm. The results were expressed as relative NAO fluorescence intensity per 1 million cells. 

### 4.7. Lipid Extraction

Lipid extraction from RPTEC cell cultures was performed using a modified Folch method [59]. Cells were counted and mixed with 4 mL chloroform, 2 mL methanol, and 1 mL deionized water (4:2:1 vol.). An internal standard (CL (C14:0)_4_) was added into the mixture at a final concentration after extraction of 40 µg/mL. The mixture was vortexed and centrifuged at 2000 rpm for five minutes at room temperature. After centrifugation, the lower chloroform-lipid phase was transferred into a glass test tube and placed under a nitrogen stream for evaporation. The following steps were repeated three times: the remaining methanol phase was mixed with 2 mL chloroform, vortexed, and centrifuged at 2000 rpm for 5 min, then the lower chloroform-lipid phase was transferred to the same glass test tube for evaporation. After the chloroform evaporated, the lipids were resuspended in 300 µL chloroform: 2-propanol mixture (1:5 vol.), filtered through a 22 µm syringe filter into a dark glass chromatographic vial. The prepared samples were analyzed immediately or stored at −80 °C. 

### 4.8. Cardiolipin Analysis Using UPLC-MS/MS

Separation and investigation of individual cardiolipin forms was performed by ultra-effective liquid chromatography—tandem mass spectrometry (UPLC-MS/MS) using Waters Acquity UPLC chromatography system coupled with a Xevo TQD (triple quadrupole) mass spectrometer (Waters, Milford, MA, USA) as described before [60]. Data was analyzed using MassLynx V4.1. software (Waters).

Mass spectrometric analysis of different cardiolipin forms in RPTEC cells was performed based on previously performed rat kidney mitochondrial cardiolipin analysis after confirming typical cardiolipin fragmentation products (fatty acid and diacylglycerol fragments) and assigning chromatographic retention factors—cardiolipin retention times in relation to internal standard retention time CL (C14:0)_4_ (Table 1). Cardiolipin quantification was based on chromatographic peak area integration standardized to peak area of internal standard and cell number used for lipid extraction. Individual cardiolipin amounts were expressed in relative units per 1 million cells. 

### 4.9. Gene Expression Analysis Using RT-qPCR

Total RNA from RPTEC cells was extracted by using TRIzol reagent (Thermo Fisher Scientific) following manufacturer’s protocol. Briefly, RPTEC cells were mixed with 0.5 mL TRIzol and incubated for 5 min for complete dissociation of nucleoproteins. Then, 0.1 mL chloroform was added, mixed well by vortexing, incubated for 2–3 min and centrifuged for 15 min at 12,000× *g*, at 4 °C. Aqueous phase was separated and mixed with 0.25 mL isopropanol and incubated for 10 min, then centrifuged for 10 min at 12,000× *g*, at 4 °C. Supernatant was removed, and RNA pellet was washed by resuspending with 0.5 mL 75% ethanol, vortexed and centrifuged for 5 min at 7500× *g*, at 4 °C. The RNA washing step was performed twice. After washing RNA pellet the supernatant was removed, the pellet was air dried for 5–10 min, resuspended in 40 µL RNase-free water and stored in −80 °C until analysis. The total RNA yield was determined spectrophotometrically by using Nanophotometer Pearl (Implen, Munich, Germany).

Total RNA was reverse transcribed using High-Capacity cDNA Reverse Transcription Kit (Applied Biosystems, Waltham, MA, USA) and 600 ng of total RNA was used per reaction. Gene expression levels were measured using TaqMan gene expression assays (Assay IDs: *CRLS1* Hs00219512_m1, *TAZ* Hs00902887_g1; *LCLAT1* Hs00699427_m1) following manufacturer’s protocol using Quiagen Rotor Gene Q 5plex HRM real-time PCR cycler (QIAGEN, Thermo Fisher Scientific, USA). The cycle threshold (Ct) values were normalized to the value of *HPRT1* (Assay ID: Hs99999909_m1) reference gene. Data was analyzed using Rotor-Gene Q Series Software version 2.3.1. Relative gene expression was quantified using 2^−ΔΔCT^ method. 

### 4.10. Analysis of Cardiolipin Synthesis/Remodeling Enzyme Levels in RPTEC Cells

Cardiolipin synthase levels were analyzed in RPTEC cell lysates using Human Cardiolipin Synthase (CRLS1) ELISA Kit (abx253754, abbexa, Cambridge, UK). Lysocardiolipin acyltransferase (LCLAT1) levels were analyzed in RPTEC cell lysates using Human Lysocardiolipin Acyltransferase 1 (LCLAT1) ELISA Kit (abx530801, abbexa). Tafazzin levels were analyzed in RPTEC cell lysates using Human Tafazzin ELISA kit (A7576, antibodies.com, Cambridge, UK). All assays were performed following manufacturers’ protocols. The results were normalized to cell protein levels by using the Bradford method. 

### 4.11. Statistical Analysis

The data were presented as means ± SE. If the data were normally distributed (Shapiro-Wilk test of normality) statistical analysis was performed using ANOVA followed by post-hoc LSD or Dunnet’s T3 test (for non-equal variances, by Levene’s test), or independent-samples Student’s *t*-test. Alternatively non-parametric tests were used. Analysis was performed using IBM SPSS Statistics 29.0.1.0 (IBM, Chicago, IL, USA) and MS Excell software (version 2404, Microsoft, Redmond, WA, USA). *p* < 0.05 was considered significant.

## 5. Conclusions

In this study, we show that hypoxia/reoxygenation alone can induce the gene expression and synthesis of respective enzymes involved in cardiolipin synthesis and remodeling, which, in turn, elevates total cardiolipin levels in human renal proximal tubule epithelial cells. An analysis of individual cardiolipin species revealed that the amounts of most cardiolipin species significantly increased already after 24 h of hypoxia and started to decrease after 24 h of reoxygenation but remained higher than control levels. The biggest increase after hypoxia was among cardiolipins with highly unsaturated and long-chain fatty acids (C20:3, C20:4, C22:4, C22:6), and although these species, after reoxygenation, substantially decreased, their levels remained significantly higher than in the control group. Such alterations in cardiolipin amounts occurred due to increased gene expression, along with increased protein levels of cardiolipin synthase and lysocardiolipin acyltransferase (LCLAT1, also known as ALCAT1). These changes in cardiolipin levels were accompanied by a significant reduction in mitochondrial respiration after 24 h of hypoxia that was not restored after 24 h of reoxygenation. In addition, the rate of ROS production was significantly elevated after 24 h of reoxygenation. Therefore, this study provides new insights into the cardiolipin content of human renal proximal tubule epithelial cells, as well as the impact of hypoxia and reoxygenation on cardiolipin metabolism in these cells. 

## Figures and Tables

**Table 1:** Individual cardiolipin species and proposed fatty acid composition based on cardiolipin mass and fragmentation in mass spectrometry.

Proposed CL FA Composition	CL Mass, *m*/*z*	Diacylglycerol Phosphate Fragment, *m*/*z*	Fatty Acid Fragment, *m*/*z*	Retention Factor (CL Retention Time/IS Retention Time)
(18:1)(18:2)(16:0)(16:1)	1400	671	281, 253	1.16
(C18:2)_3_(C16:1)	1422	695	279, 253	0.99
(C18:2)_2_(C18:1)(C16:1)	1424	669, 671, 697	279, 253	1.06
(18:2)(18:1)_2_(16:1)	1426	671, 697, 728	253	1.15
(18:2)_2_(18:1)(16:0)	1426	671, 695	279, 255	1.16
(18:2)(18:1)_2_(16:0)	1428	697	281, 279, 255	1.23
(18:2)_4_	1448	695	279	0.995
(18:2)_3_(18:1)	1450	697	279, 281	1.08
(18:2)_2_(18:1)_2_	1452	695	281, 279	0.998
(18:2)_3_(18:0)	1452	695, 699	283, 279	1.07
(18:2)_3_(20:4)	1472	695, 719	279, 303	0.92
(18:2)_2_(18:1)(20:4)	1474	719	279, 281, 303	0.98
(18:2)_2_(C18:0)(20:3)	1476	723, 699, 695	279, 283, 303	0.981
(18:2)_3_(22:6)	1496	695	279, 327	0.98
(22:6)(18:1)_2_(18:2)	1500	697	279, 281, 327	1.057
(22:4)(20:3)(16:1)(18:1)	1502	721, 723	281, 305, 331	1.08

## Data Availability

The data that support the findings of this study are available from the corresponding author upon request.
